# The Src-Kinase Fyn is Required for Cocaine-Associated Memory Through Regulation of Tau

**DOI:** 10.3389/fphar.2022.769827

**Published:** 2022-02-03

**Authors:** Hongchun Li, Xinglong Zhou, Rong Chen, Yuzhou Xiao, Tao Zhou

**Affiliations:** ^1^ National Chengdu Center for Safety Evaluation of Drugs, State Key Laboratory of Biotherapy/Collaborative Innovation Center for Biotherapy, West China Hospital, Sichuan University, Chengdu, China; ^2^ Department of Respiratory and Critical Care Medicine, Targeted Tracer Research and Development Laboratory, West China Hospital, Sichuan University, Chengdu, China; ^3^ Department of Drug and Equipment, China Rongtong Bayi Orthopaedic Hospital, Chengdu, China

**Keywords:** Fyn, cocaine, Tau, drug addicion, PP2

## Abstract

Drug-associated context-induced relapse of cocaine-seeking behaviour requires the retrieval of drug-associated memory. Studies exploring the underlying neurobiological mechanism of drug memory formation will likely contribute to the development of treatments for drug addiction and the prevention of relapse. In our study, we applied a cocaine-conditioned place preference (CPP) paradigm and a self-administration paradigm (two drug-associated memory formation model) to confirm the hypothesis that the Src kinase Fyn critically regulates cocaine-associated memory formation in the hippocampus. For this experiment, we administered the Src kinase inhibitor PP2 into the bilateral hippocampus before cocaine-CPP and self-administration training, and the results showed that pharmacological manipulation of the Src kinase Fyn activity significantly attenuated the response to cocaine-paired cues in the cocaine-CPP and self-administration paradigms, indicating that hippocampal Fyn activity contributes to cocaine-associated memory formation. In addition, the regulation of cocaine-associated memory formation by Fyn depends on Tau expression, as restoring Tau to normal levels disrupted cocaine memory formation. Together, these results indicate that hippocampal Fyn activity plays a key role in the formation of cocaine-associated memory, which underlies cocaine-associated contextual stimulus-mediated regulation of cocaine-seeking behaviour, suggesting that Fyn represents a promising therapeutic target for weakening cocaine-related memory and treating cocaine addiction.

## Introduction

Drug addiction is a prevalent neuropsychiatric disease that incurs high financial costs to society resulting from the potential for chronic relapse (Barry and McGinty, 2017). Thus, relapse prevention is a particularly important goal for treatment. Long-lasting drug-associated memory formation is closely associated with the rewarding effects of the drug and drug exposure context (Bender and Torregrossa, 2020). Accumulating preclinical studies have also suggested that exposure to drug-associated contextual stimuli may reactivate drug-associated memories, evoke craving, and promote drug relapse in drug addicts (Farrell et al., 2018). Therefore, a better understanding of the underlying mechanism of drug-associated memory formation is necessary for the development of treatments.

The hippocampus, an important brain region and component of addiction circuitry, plays a critical role in drug-context memory encoding (Lehmann and Kenny, 2020). For example, the hippocampus is involved in the formation of cocaine-associated memories that directly induce cocaine-seeking behaviour through connections with the basolateral amygdala (Wells et al., 2011). However, the mechanism by which the hippocampus regulates drug-associated memory formation, especially the underlying neurobiological mechanism, has not been completely elucidated. Thus, studies exploring the intracellular signalling pathways in the hippocampus that affect cocaine-associated memory formation may provide new therapeutics for relapse prevention.

Tau, a microtubule-associated scaffolding protein, was shown to be necessary for drug-associated memory formation in our previous study (Li et al., 2021). In our previous research, we first found that Tau expression is significantly downregulated in the hippocampus during cocaine-associated memory formation, promoting adult hippocampal neurogenesis in the hippocampal dentate gyrus and facilitating enhanced cocaine-associated memory formation (Li et al., 2021). Nevertheless, the mechanism underlying the reduction in Tau levels during cocaine-associated memory formation remains unclear. Based on previous accumulating evidence, Tau levels are critically regulated by the Src kinase Fyn (Chen et al., 2013; Li and Gotz, 2017). For example, the Fyn/ERK/S6 signalling pathway mediates the *de novo* synthesis of the Tau protein in the somatodendritic compartment (Li and Gotz, 2017). Treatment with the Fyn pathway inhibitor PP2 reverses the prion protein (PrPC)-induced reduction in Tau levels, suggesting that the Fyn pathway may have an important role in regulating Tau levels (Chen et al., 2013). Additionally, activation of the Src kinase Fyn in the dorsal striatum and hippocampus contributes to drug-induced behavioural phenomena, such as drug-primed ethanol-seeking (Wang et al., 2010) and context-elicited cocaine-seeking behaviours (Xie et al., 2013), respectively. Therefore, the Tau level-dependent putative contributions of Fyn expressed in the hippocampus to cocaine-associated memory formation have not been explored.

In our study, we aimed to evaluate whether the Src kinase Fyn activity within the hippocampus is necessary for the formation of cocaine-associated memory that promotes cocaine context-induced seeking behaviour and whether the underlying mechanism depends on the regulation of Tau expression. Our results suggest that cocaine-associated memory formation is mediated by the Src kinase Fyn and that Fyn inhibitors are potentially promising treatments for cocaine addiction.

## Materials and Methods

### Animals

Wild-type (WT) male C57BL/6J mice (8–12 weeks old) were purchased from Vital River Laboratory Animal Technology Co., Ltd. (Beijing, China). Tau^-/-^ knockout (KO) mice were obtained from Jackson Laboratories (#007251, United States), and male Tau^-/-^ mice were used in our study. All mice were housed in an animal facility on a standard 12-h light/12-h dark cycle (lights on from 7:00 A.M. to 7:00 P.M.) with *ad libitum* access to food and water at room temperature (22–28°C). All experimental procedures and animal protocols were performed in accordance with guidelines from the Institutional Animal Care and Use Committee of West China Hospital of Sichuan University (20211398A). All efforts were made to minimize the suffering of the mice.

### Drugs

Cocaine was obtained from the National Institute for the Control of Pharmaceutical and Biological Products (Beijing, China) and dissolved in saline. PP2 (S7008, Selleck) was dissolved in vehicle (saline containing 1% dimethylsulfoxide).

### Conditioned Place Preference

The CPP paradigm was performed using a standard three-chambered apparatus comprising two large conditioning compartments (black and white) with different floors (bar and grid floors) and a small middle chamber (grey, smooth PVC floor) connecting the two large compartments. Before each session, the animals were habituated to the chambers for at least 10 min on 2 consecutive days. During the pretest session, mice were placed in the chamber and provided free access to all chambers for 15 min, and the time spent in each chamber was recorded. After the pretest, animals that represented a strong unconditioned preference for either chamber (chamber bias, >300 s) were excluded, and about 50 animals were excluded from the CPP experiments in our study. The remain animals were randomly assigned to two groups, one group that received alternating injections of cocaine [20 mg/kg, intraperitoneal (i.p.)] and saline and the other group that received saline injections in both compartments, and trained for 6 days. After the injection, animals were trained in the conditioning or nonconditioning chambers for 30 min and then returned to their home cages. On the test day, the mice were placed in the neutral chamber and allowed to explore both compartments for 15 min. The time spent in each chamber was measured to calculate the CPP score. CPP scores are recorded as the time spent in the cocaine-paired chamber minus the time spent in the saline-paired chamber.

### Cocaine Self-Administration

Mice were anaesthetized with sodium pentobarbital (60 mg/kg), and a single sterilized silastic catheter (0.51 ID x 0.94 mm OD, BB518-20, Scientific Commodities) was implanted into the right jugular vein. The skin on the animal’s back was attached to the distal end of the catheter *via* a stainless steel guide cannula (RWD Life Science). After surgery, mice received 0.1 ml of a saline solution containing penicillin (160000/ml) and heparin (30 U/ml) via the catheters daily. Following 1 week of recovery, the animals were trained to intravenously self-administer cocaine (0.75 mg/kg/infusion) or saline in daily 2 h sessions over 10 days on an FR1 schedule in an operant chamber. Active pokes resulted in the injection of cocaine accompanied by the presentation of blue light for 20 s and an audible tone for a 5 s timeout period. Inactive pokes failed to elicit drug injection and presentation of the conditioning stimuli.

### Locomotor Activity

Locomotor activity was recorded as the distance travelled. The animals were acclimated to chambers (48 cm × 48 cm) for 10 min on 2 consecutive days. Baseline locomotor activity was not significantly different between groups. During the injection course, animals were administered cocaine (20 mg/kg, i.p.) or an equal volume of saline and immediately placed in the chamber for 15 min. The distance travelled was recorded daily for 7 consecutive days, and automated tracking and recording were performed with EthoVision 7.0 software (EthoVision 7.0; Noldus Information Technology, Leesburg, VA).

### Y-Maze Test

Y-mazes are often used to assess spatial memory. The experiment was performed using a maze shaped like the letter “Y”, with a starting arm and two target arms. The luminance of overhead white lighting is 40 lux and numerous visual cues were placed on the wall of the testing room. On the test day, mice were put at the end of one arm (designated as the start arm), and were trained to leave the start arm and enter the right arm (familiar arm), not the left arm (novel arm) for 10 min. After 120 min inter-trial interval, the novel arm was opened, the mice were allowed to explore all three arms. The choices of novel and familiar arms were alternate between tests of different subjects, and the time spent in the novel and familiar arms during the 5-min test was automatically recorded by the software (ANYmaze, United States).

### Novel Object Position Experiments

Animals were placed in a test chamber (48 cm × 48 cm) for 10 min on the first day (without objects). On the second day, mice were placed in the test chamber for 10 min (with two identical objects). On the third day, the familiar object was shifted to a diagonal position (different position but the same size and colour as the familiar object). The animals were placed again in the area and allowed to explore the object at the novel position for 10 min. The time spent exploring the object was recorded by EthoVision 7.0 software (EthoVision 7.0; Noldus Information Technology, Leesburg, VA).

### Novel Object Recognition Experiments

The mice were placed in a test chamber (48 cm × 48 cm) for 10 min on the first day (without objects). On the second day, mice were placed in the test chamber for 10 min (with two identical objects). On the third day, one of the familiar objects was replaced with a novel object (different size and colour from the familiar object). Animals were again placed in the area and allowed to explore the novel object for 10 min. The time spent exploring the object was recorded by EthoVision 7.0 software (EthoVision 7.0; Noldus Information Technology, Leesburg, VA).

### Stereotaxic Surgery and Inhibitor Administration

Animals were anaesthetized with sodium pentobarbital (60 mg/kg) and mounted on a standard stereotaxic frame (RWD Life Science). The hair of each mouse was shaved, the incision site was cleaned with medical alcohol, the scalp was incised, the skull was exposed, and the hippocampus (AP, −1.7 mm; ML, ±1.2 mm; DV, −1.5 mm) was bilaterally implanted with permanent guide cannulas (RWD Life Science) using a stereotaxic instrument. The guide cannula was anchored with dental cement, and a stainless-steel stylet blocker was inserted into each cannula to prevent blockage and infection. All mice were subjected to training after 1 week of recovery.

PP2, an ATP-competitive Src kinase inhibitor, was dissolved in saline containing 1% DMSO at a final concentration of 62.5 ng/μL. PP2 was injected bilaterally (1 μL/side, 0.5 μL/min) with a microinjector 15 min before cocaine or saline administration.

### Lentiviral Vector Construction

LV-pClenti-hSyn-EGFP-3xFLAG-WPRE was purchased from Obio Technology Co., Ltd. (Shanghai, China). The Fyn shRNA was cloned into pClenti-hSyn-EGFP-3xFLAG-WPRE and confirmed by sequencing. The sequences of the scrambled control shRNA and Fyn shRNA were 5′-TTC​TCC​GAA​CGT​GTC​ACG​T-3′ and 5′-CCC​AAG​AGG​TAC​CTT​TCT​T-3′, respectively. Recombinant lentiviruses were produced by transient transfection in HEK293T cells, and then the level of Fyn was analysed using western blot.

### Stereotaxic Injection of LV-shRNA-Fyn in the Hippocampus

Mice (8–12 weeks old) were anaesthetized with sodium pentobarbital (60 mg/kg) and placed on a stereotaxic apparatus (RWD Life Science) to inject the virus into the dorsal hippocampus (AP, −1.7 mm; ML, ±1.2 mm; DV, −1.5 mm). After shaving the hair and cleaning the incision site with medical grade alcohol, the scalp was incised to expose the skull, and the connective tissue was gently removed from the skull surface with cotton swabs. Small craniotomy holes were drilled with a skull rotor (RWD Life Science) for virus injection. Microsyringe needles were used to bilaterally infuse the hippocampal tissue with 1 μL of virus at a rate of 0.1 μL/min. After each injection, the syringe was left in place for an additional 5 min and then slowly withdrawn to allow the virus to diffuse. Mice recovered for at least 1 week before behavioural testing.

### Western Blot Analysis

Hippocampal tissues were lysed, and proteins were extracted using a protein extraction kit (K269-500, Biovision). A Bradford assay (P0006, Beyotime) was used to determine the total protein concentration. Twenty micrograms of protein were loaded and separated on a 10% sodium dodecyl sulfate-polyacrylamide gel and then transferred to a polyvinylidene difluoride (PVDF) membrane (IPVH00010, Millipore) in a mixture of Tris-glycine buffer and 20% (v/v) methanol. The membrane was blocked with 5% non-fat dry milk for 1 h and then incubated with the primary antibody with gentle shaking overnight at 4°C. The next day, after three washes, the blots were incubated with the corresponding secondary antibody for 2 h at room temperature. Protein levels were visualized using a chemiluminescence substrate (WBKLS0500, Millipore) and a chemiluminescence imaging system (CLINX, Shanghai, China). Chemi Analysis software was used to quantify the optical density of each band (CLINX, Shanghai, China). The following antibodies were used for Western blot: mouse anti-Tau (1:1,000, Thermo Scientific), rabbit anti-Fyn (1:1,000, Abcam), rabbit anti-p-Fyn (T 416) (1:1,000, Cell Signaling Technology), rabbit anti-β-actin (1:1,000, Cell Signaling Technology), HRP-conjugated goat anti-mouse (SAB), and HRP-conjugated goat anti-rabbit (SAB).

### Haematoxylin-Eosin Staining

**FIGURE 1:**
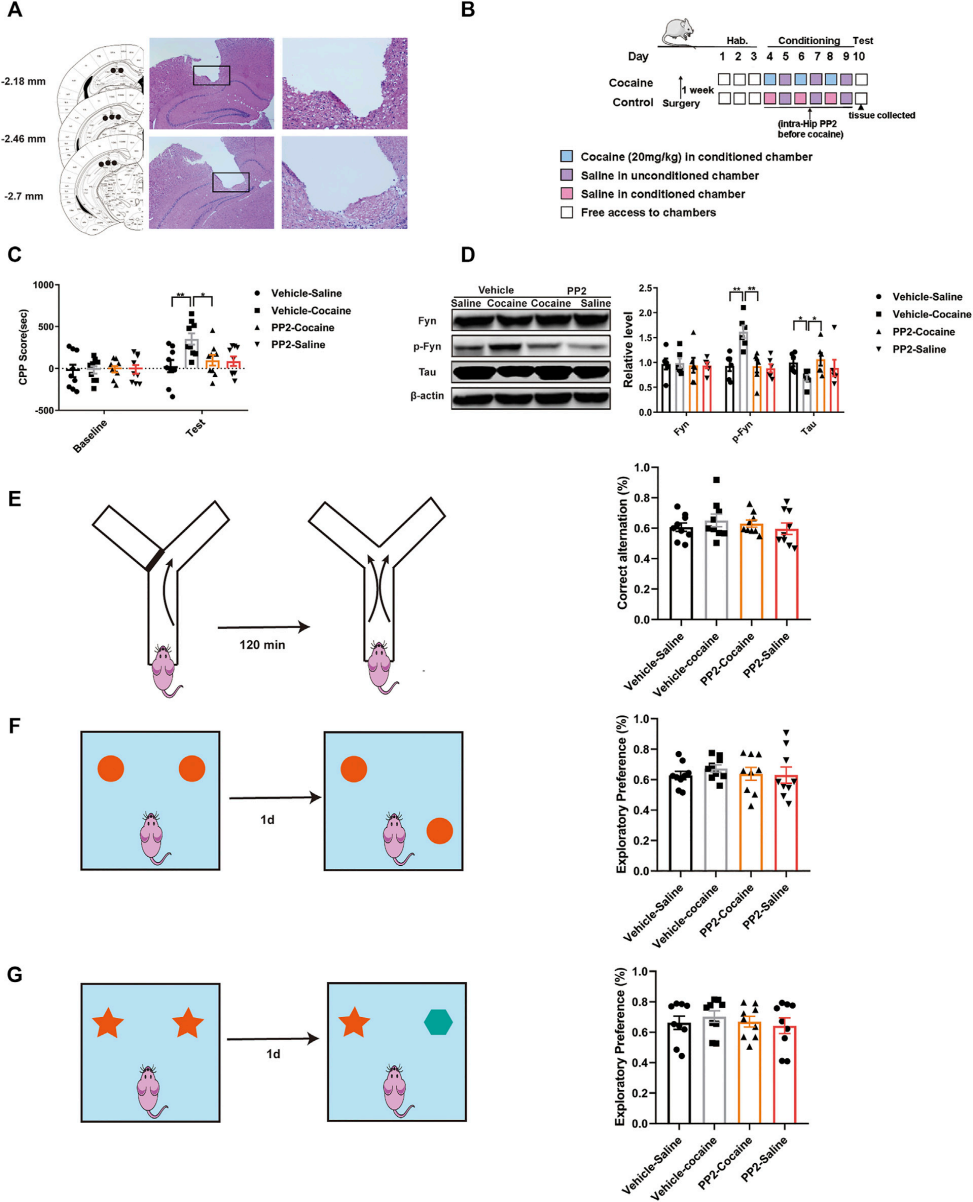
PP2 infusion into the hippocampus reduced the cocaine CPP score. **(A)** Schematic representation of the hippocampal cannula placements and injection sites. **(B)** Experimental timeline for intra-hippocampal injection of PP2 in the cocaine CPP test. **(C)** Inhibition of Fyn activity by PP2 significantly attenuated the cocaine CPP score (*n* = 9 per group). **(D)** Immunoblotting of Fyn, phosphorylated Fyn, and Tau expression in the hippocampus of cocaine CPP mice (*n* = 6 per group). **(E)** No significant differences were observed between groups in the Y-maze test (*n* = 9 per group). **(F, G)** PP2-treated mice failed to exhibiting exploratory preferences in the novel object position test and novel object recognition test (*n* = 9 per group). Data are the means ± SEM, *p < 0.05 and **p < 0.01. Hab, habituation; p, phosphorylated; Hip, hippocampus.

The cannula implantation site was confirmed by performing HE staining of 5-μm thick coronal sections, and images were captured using a light microscope (Figure 1A). Mice with misplaced cannulas were excluded from the statistical analysis.

### *In vivo* Cellular Fluorescent Labelling

Single-cell fluorescent labelling was performed as previously described with slight modifications (Luo et al., 2016). Briefly, mice (8–12 weeks old) were anaesthetized with sodium pentobarbital (60 mg/kg), placed on a stereotaxic apparatus (RWD Life Science), and the dentate gyrus received a virus injection (AP, −2.0 mm; ML, ±1.4 mm; DV, −2.2 mm). After their hair was shaved, medical-grade alcohol was used to clean the incision site, the skull was exposed via a scalp incision, and cotton swabs were used to gently remove the connective tissue from the skull surface. Small craniotomy holes were drilled with a skull drill (RWD Life Science) for virus injection. Microsyringes were used to bilaterally infuse a mixture of adeno-associated viruses (AAVs) [pAAV-Syn-DIO-(tTA-P2A-mNeonGreen)-WPRE (Obio Technology Co., Ltd., Shanghai, China) and pAAV-PTRE-tight-NLS-Cre (Obio Technology Co., Ltd., Shanghai, China)] into the brain at a rate of 0.05 μL/min for 10 min (0.5 μL per side). After each injection, the syringe was left in place for an additional 5 min and then slowly withdrawn to allow diffusion of the virus. The mice were allowed to recover for at least 3 weeks before analysing hippocampal neuron morphology.

After 3 weeks, the animals that had received the AAV injection were deeply anaesthetized with sodium pentobarbital (60 mg/kg) and perfused transcardially with phosphate-buffered saline (PBS) followed by ice-cold 4% paraformaldehyde in 0.1 M PBS (pH 7.4). We carefully extracted the brains from the skull, postfixed them with 4% PFA overnight, and then dehydrated them in 30% sucrose at 4°C. We sectioned the brains into 50 μm coronal slices using a freezing microtome (Leica, Germany), mounted them on slides, dried them and stored them at −80°C until processing for immunohistochemistry.

Sections were washed with PBS 3 times (10 min each time) and covered with anti-fade mounting medium with DAPI (H-1200, Vector). A laser confocal microscope (Nikon, Japan) was used to acquire brain images. Each neuron was scanned at high magnification (100X, oil immersion lens) to ensure that all parts of the dendrites were intact. A minimum of 3 neurons per slice from each group were examined, and at least 40 neurons were selected from each group. Confocal microscopy was performed for the 3D reconstruction of neurons. The total dendritic length and dendritic spine density were measured using ImageJ software (US National Institutes of Health, Bethesda, MD, United States).

### Transmission Electron Microscopy

Mice were deeply anaesthetized with sodium pentobarbital (60 mg/kg) by i.p. injection and 50 ml of freshly prepared fixative containing 2.5% glutaraldehyde and 2% paraformaldehyde in 0.1 M PBS (pH 7.4) were perfused intracardially. Whole brains were removed, postfixed with 2.5% glutaraldehyde overnight at 4°C, impregnated with 1% osmium tetroxide for 1 h, dehydrated in graded alcohol solutions, flat embedded in Durcupan ACM (Fluka) and cured for 48 h at 60°C. Small pieces containing the hippocampal dentate gyrus were removed from the specimens and glued on a plastic block with cyanoacrylate. Ultrathin sections were cut and mounted on Formvar-coated single-slot grids. A transmission electron microscope (JEOL, Japan) was used to observe the synapse structure. We obtained 5 images of each section, yielding at least 50–70 synapses from each mouse. Image-Pro Plus 6.0 software was used for the morphometric analysis.

### Statistical Analysis

All data were analysed with GraphPad Prism 7 software and are presented as the means ± SEMs. The normality of the data distribution was analysed using the Kolmogorov–Smirnov test. An unpaired two-tailed Student’s t test was used for simple comparisons. One-way or two-way ANOVA followed by the Bonferroni post hoc test were utilized for multiple comparisons. In all results, n refers to the animal number. For all results, statistical significance was set to *p* < 0.05.

## Results

### Fyn Inhibition Attenuates the Cocaine-CPP Score

Previous studies have suggested that PP2, a Src kinase Fyn inhibitor, is able to inhibit Fyn activity and alter Tau expression in individuals with Alzheimer’s disease (AD) (Chen et al., 2013; Li and Gotz, 2017). We explored whether inhibition of Fyn activity suppresses cocaine-associated memory formation and modulates Tau expression. The CPP paradigm, a widely used protocol for assessing drug-associated memory formation (Sanchis-Segura and Spanagel, 2006), was used to determine the role of Fyn in cocaine-associated memory formation. The timeline of the cocaine CPP procedure is shown in Figure 1B. During habituation to the CPP apparatus, neither group showed a side preference. In the training phase, cocaine induced a significant increase in the CPP score, and pre-administration of PP2 into the hippocampus significantly decreased the cocaine-CPP score [F _(3, 32)_ = 4.795, *p* < 0.01, two-way ANOVA, Figure 1C]. Injection of PP2 significantly reduced the level of phosphorylated Fyn, accompanied by an increase in Tau expression after cocaine-CPP training [p-Fyn: F _(3, 20)_ = 10.05, *p* < 0.01, one-way ANOVA; Tau: F _(3, 20)_ = 4.354, *p* < 0.05, one-way ANOVA, Figure 1D]. In addition, to assess whether PP2 altered the working and spatial memory of mice after cocaine-associated memory formation, we also assessed short-term working memory and short-term spatial and recognition memory using the Y-maze, novel object position and recognition test after cocaine-CPP training, respectively. No significant PP2-induced impairment in the spontaneous alternation behaviour was observed in the Y-maze tests (Figure 1E). Compared with control groups, PP2-treated mice displayed no preference for the novel location of an object during the novel object position test, indicating no deficit in the spatial memory after PP2 administration (Figure 1F). Similarly, in the novel object recognition test, PP2 did not reduce the exploratory behaviour or preference for novel objects (Figure 1G). Based on these results, pharmacological blockade of Fyn activity impairs cocaine-associated memory and is accompanied by increased Tau expression, rather than affecting short-term working memory and short-term spatial and recognition memory.

### Fyn Inhibition Prevents Cocaine Self-Administration

**FIGURE 2:**
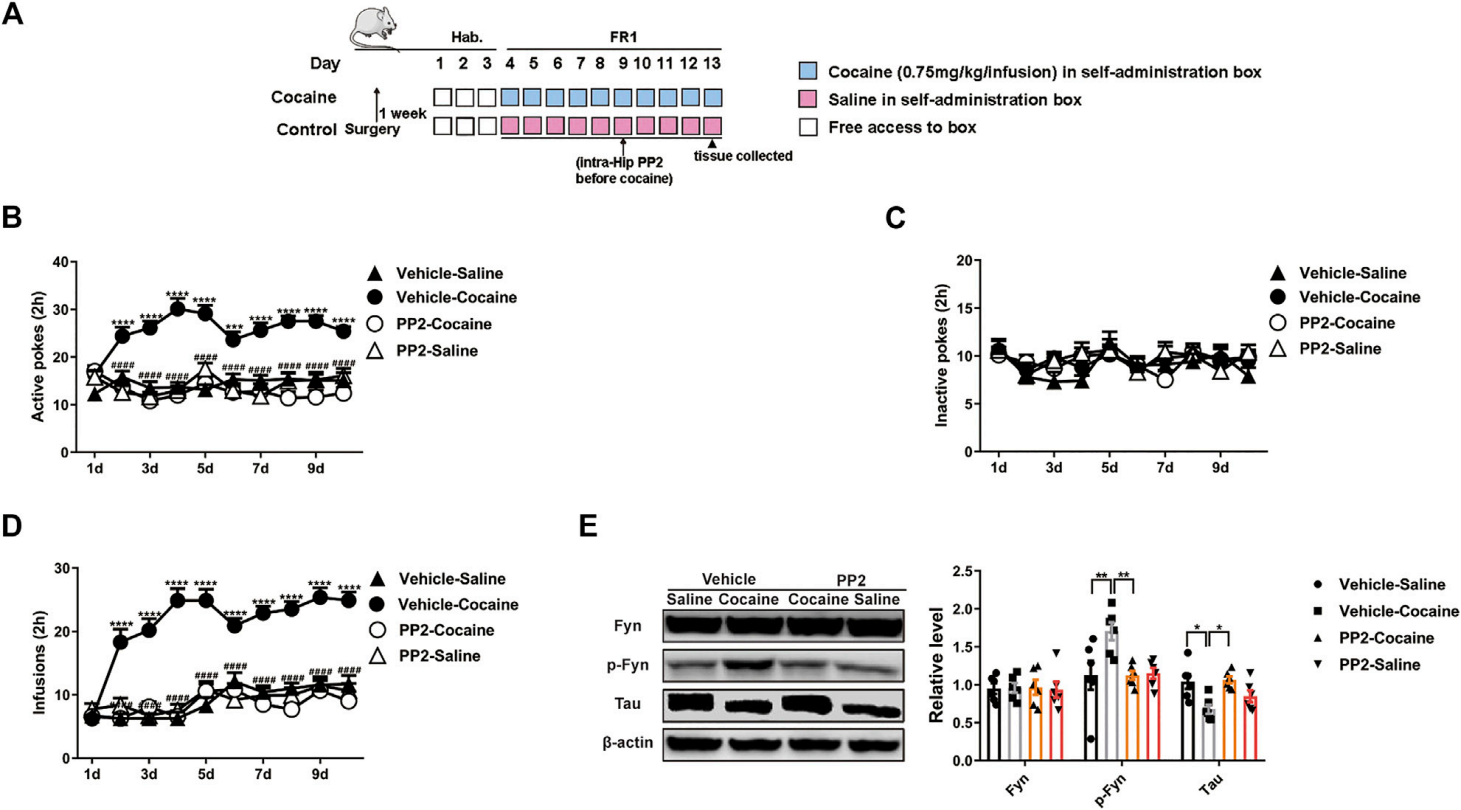
PP2 infusion into the hippocampus disrupts cocaine self-administration. **(A)** Procedure of cocaine self-administration experiment. **(B–D)** Number of active pokes, inactive pokes, and infusions in cocaine self-administration (*n* = 10 per group). **(E)** Levels of Fyn, phosphorylated Fyn, and Tau in the hippocampus of cocaine self-administered mice (*n* = 6 per group). Data are the means ± SEM, *p < 0.05; **p < 0.01; ***p < 0.001, and ****p < 0.0001.

We continued to examine the role of Fyn in cocaine self-administration, a paradigm that incorporates memories of the rewarding effects of the drug and drug exposure context. Cocaine-associated memory is formed when an instrumental action (an active poke) results in cocaine injection (the unconditioned stimulus) and is paired with an audiovisual cue (the conditioned stimulus). The mice were trained on an FR1 schedule of reinforcement, in which a single active poke induced an infusion of cocaine or saline. The timeline of the cocaine self-administration procedure is shown in Figure 2A. Compared with saline-treated mice, the cocaine-treated mice showed a marked increase in the number of active pokes. Thus, the cocaine-treated mice exhibited strong and reliable cocaine-associated memory formation in the self-administration paradigm (Figure 2B). Moreover, we also found that the number of active pokes decreased after the administration of PP2 (Figure 2B). In contrast, the numbers of active pokes and inactive pokes were not different between vehicle- and PP2-pretreated mice during saline treatment [active pokes: F _(27, 360)_ = 3.497, *p* < 0.0001, repeated measures two-way ANOVA, Figures 2B,C]. In addition, the number of cocaine infusions increased during cocaine-associated memory formation, and PP2 pre-treatment decreased the number of cocaine infusions [F _(27, 324)_ = 5.800, *p* < 0.0001, repeated measures two-way ANOVA, Figure 2D]. Finally, PP2 also obviously decreased the levels of phosphorylated Fyn and reversed the downregulation of Tau expression in the hippocampus of the self-administration paradigm [p-Fyn: F _(3, 20)_ = 5.604, *p* < 0.01, one-way ANOVA; Tau: F _(3, 20)_ = 4.814, *p* < 0.05, one-way ANOVA, Figure 2E]. Collectively, our results show that PP2 may reduce cocaine-induced seeking behaviours in a self-administration paradigm.

### Fyn Inhibition Fails to Block Cocaine-Induced Locomotor Activity

**FIGURE 3:**
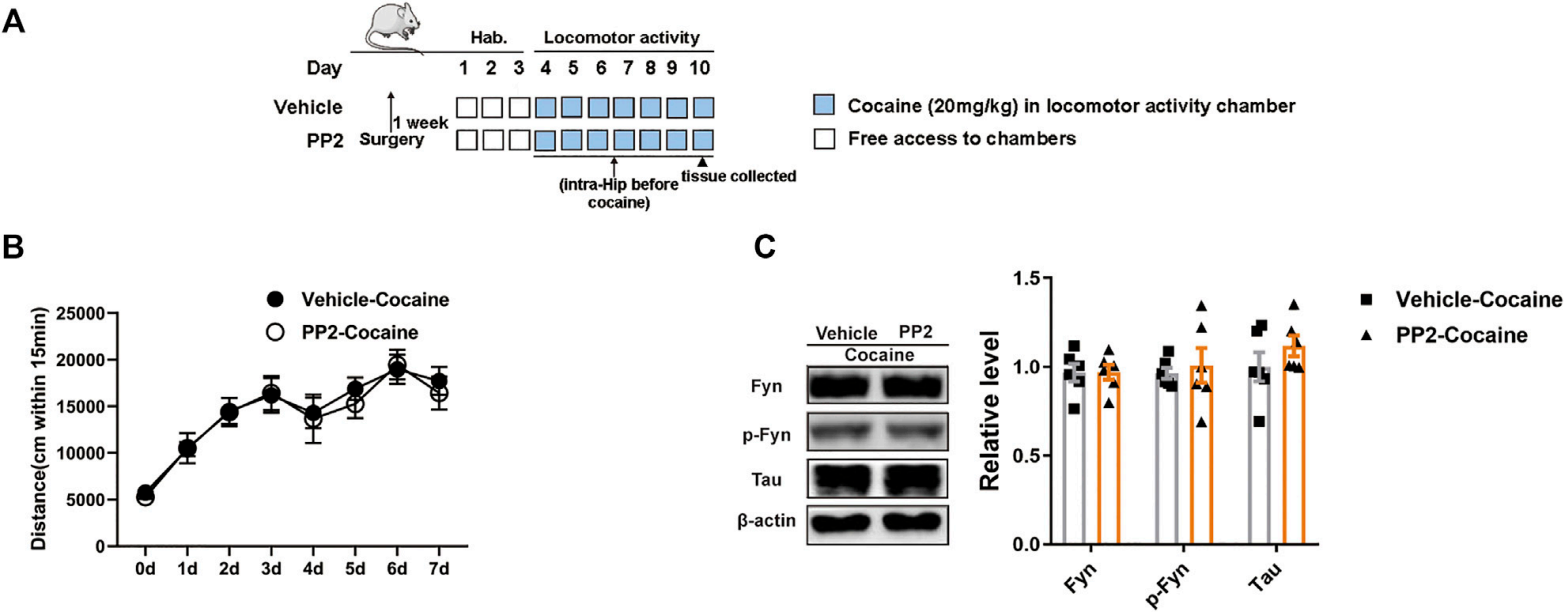
PP2 infusion into the hippocampus failed to weaken cocaine locomotor activity. **(A)** Timeline of cocaine locomotor-activity. **(B)** Total distance traveled 15 min after daily cocaine administration (*n* = 10 per group). **(C)** Western blot of Fyn, phosphorylated Fyn, and Tau in the hippocampus after cocaine hyperlocomotion paradigm (*n* = 6 per group). Data are the means ± SEM.

A locomotor activity assay in which cocaine-associated contextual stimuli were absent was used to measure the psychomotor and psychostimulant effects of cocaine by assessing the distance travelled after drug exposure. The timeline of the cocaine locomotor activity procedure is shown in Figure 3A. Compared to the cocaine group, the PP2 pre-treatment group did not exhibit a significant decrease in distance travelled (Figure 3B). In addition, PP2 did not alter the hippocampal levels of phosphorylated Fyn or Tau during cocaine-induced hyperlocomotion (Figure 3C). Therefore, cocaine-induced hyperlocomotion is not attenuated by PP2, further indicating that the Src kinase Fyn is involved in cocaine-associated memory formation through regulation of Tau levels in the hippocampus.

### PP2 Alters Dendrite Structural Remodelling and the Synapse Number in Hippocampal Neurons After Cocaine CPP

**FIGURE 4:**
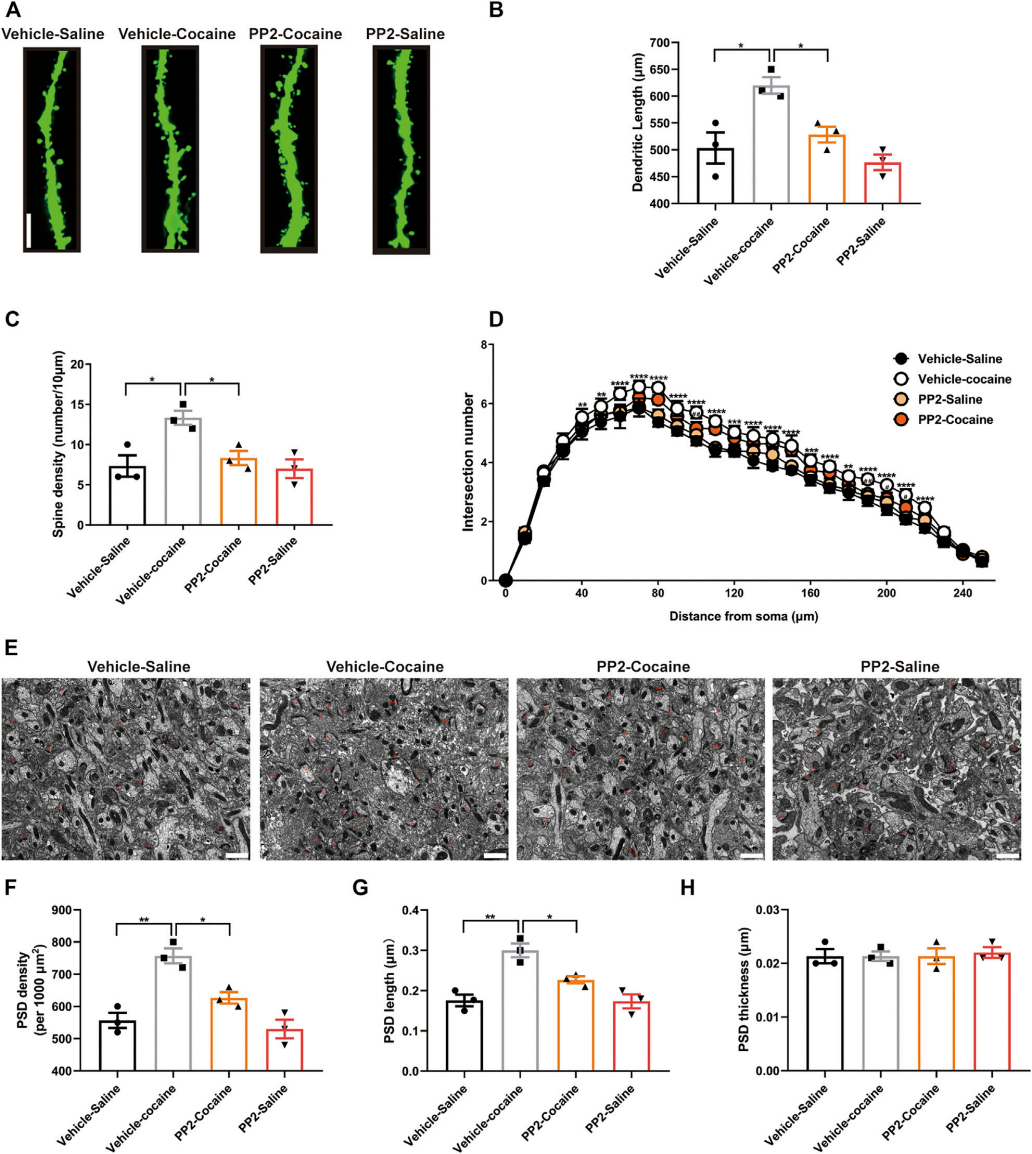
Cocaine remodels dendrite structure and synapse numbers in hippocampal neurons depending on Fyn activity. **(A)** Representative images of dendritic spines in hippocampal neurons (100 × oil lens), Scale bars: 10 μm. **(B)** PP2 blockade reduced the dendritic length promoted by cocaine (*n* = 3 per group). **(C)** PP2 blockade obviously attenuated the spine density promoted by cocaine (*n* = 3 per group). **(D)** PP2 inhibited cocaine-induced dendritic arborization (*n* = 3 per group). **(E)** Representative images of the morphology of synapses in the dorsal dentate gyrus region under electron microscopy (*n* = 3 per group). **(F–H)** PSD density and length, but not thickness, were decreased in the hippocampal dorsal dentate gyrus after PP2 pretreatment, as determined by electron microscopy analysis. The red arrowheads indicate synapses. Scale bars: 1 μm. Data are the means ± SEM, *p < 0.05 and **p < 0.01.

Previous studies have shown that cocaine-induced structural changes in neurons are involved in cocaine-mediated behavioural adaptations (Cahill et al., 2016). We explored whether PP2 suppresses cocaine memory formation by remodelling dendritic structures and altering synapse numbers by assessing the effect of PP2 on the dendritic structure of hippocampal granule neurons in mice subjected to cocaine-CPP training. We analysed the dendritic complexity of granule neurons in the dorsal dentate gyrus, which generally exhibits adult hippocampal neurogenesis, and promotes memory formation (Aimone et al., 2006), using ImageJ software and the Sholl analysis plugin, as described previously (Xu et al., 2020). In our study, cocaine significantly increased the dendritic complexity, including increasing the dendritic length and spine density (Figures 4A–D). PP2 administration inhibited the cocaine-induced increase in dendritic length and obviously reversed the cocaine-induced increase in spine density [Dendritic length: F _(3, 8)_ = 10.29, *p* < 0.05, one-way ANOVA; Spine density: F _(3, 8)_ = 7.429, *p* < 0.05, one-way ANOVA, Figures 4B,C]. In addition, PP2 reduced cocaine-induced dendritic arborization after cocaine-CPP training [F _(75, 208)_ = 2.393, *p* < 0.0001, repeated measures two-way ANOVA, Figure 4D]. We also performed electron microscopy to analyse synaptic alterations during cocaine-associated memory formation. An analysis of dorsal dentate gyrus synapses in cocaine-treated mice revealed significant increases in the density and length of the postsynaptic density (PSD) after cocaine-CPP training (Figures 4E–G), whereas no alteration in the thickness (a measure of maturation) of the PSD was observed between the cocaine-treated group and the control group (Figure 4H). However, this increase was obviously attenuated by the PP2 injection [PSD density: F _(3, 8)_ = 18.55, *p* < 0.01, one-way ANOVA; PSD length: F _(3, 8)_ = 15.72, *p* < 0.01, one-way ANOVA, Figures 4E–G]. Thus, our studies suggest that Fyn activation contributes to cocaine-induced dendritic remodelling of granule neurons and increases the number of synapses in the hippocampus after cocaine-CPP training.

### Fyn Inhibition Decreases the Cocaine-CPP Score in a Tau-Dependent Manner

**FIGURE 5:**
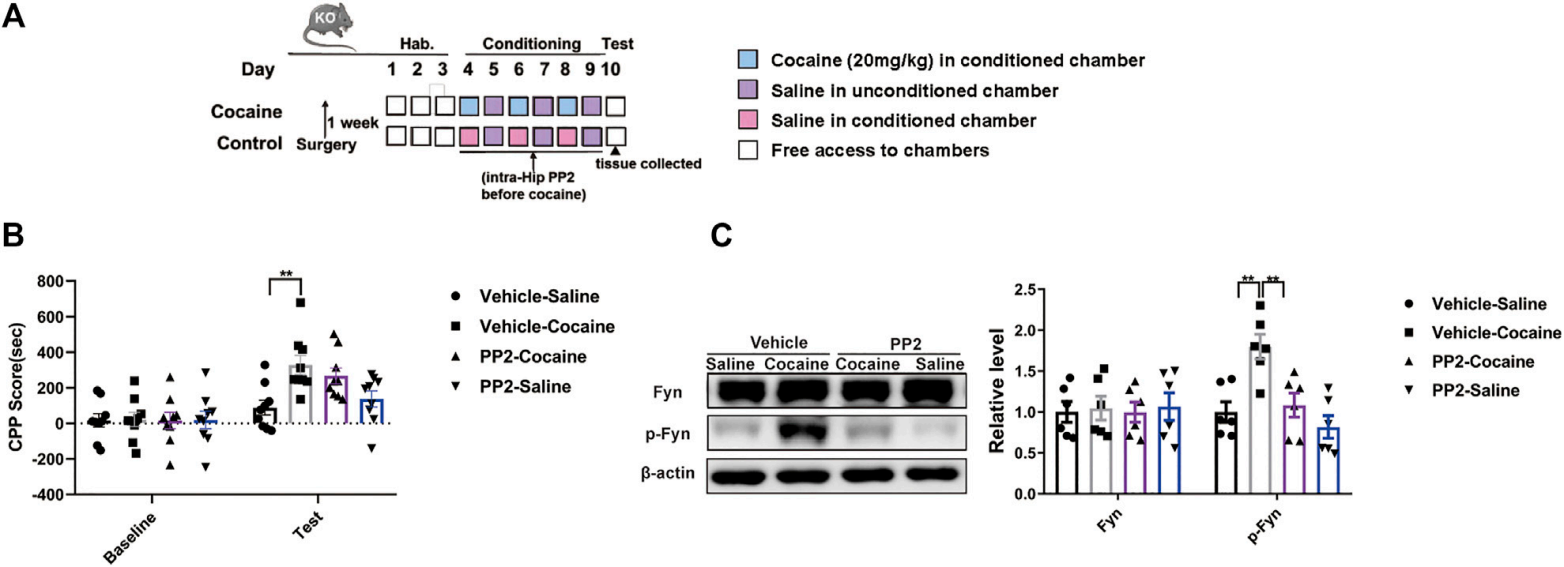
Fyn inhibition weakens the cocaine CPP score depending on Tau expression. **(A)** Experimental timeline for PP2 injection in the cocaine CPP test of Tau-KO mice. **(B)** Inhibition of Fyn activity by PP2 unable significantly attenuated the cocaine CPP score (*n* = 9 per group). **(C)** Immunoblotting of Fyn and phosphorylated Fyn expression in the hippocampus of Tau-KO mice (*n* = 6 per group). Data are the means ± SEM, **p < 0.01.

We injected PP2 into the hippocampus of Tau KO mice during CPP training to further confirm whether the role of Fyn in regulating cocaine-associated memory formation depends on Tau expression. The timeline of the cocaine CPP procedure is shown in (Figure 5A). Compared with saline-injected Tau KO mice, cocaine-injected Tau KO mice showed a marked increase in the CPP score (Figure 5B). PP2 pre-treatment failed to affect the cocaine-CPP score of Tau KO mice, although the level of phosphorylated Fyn was decreased [CPP Score: F _(3, 32)_ = 5.762, *p* < 0.01, two-way ANOVA; p-Fyn: F _(3, 20)_ = 9.356, *p* < 0.01, one-way ANOVA, Figures 5B,C]. Collectively, these results provide strong evidence that Fyn modulates cocaine memory formation, possibly by depending on Tau expression.

### Downregulation of the Fyn Level Attenuates the Cocaine-CPP Score

**FIGURE 6:**
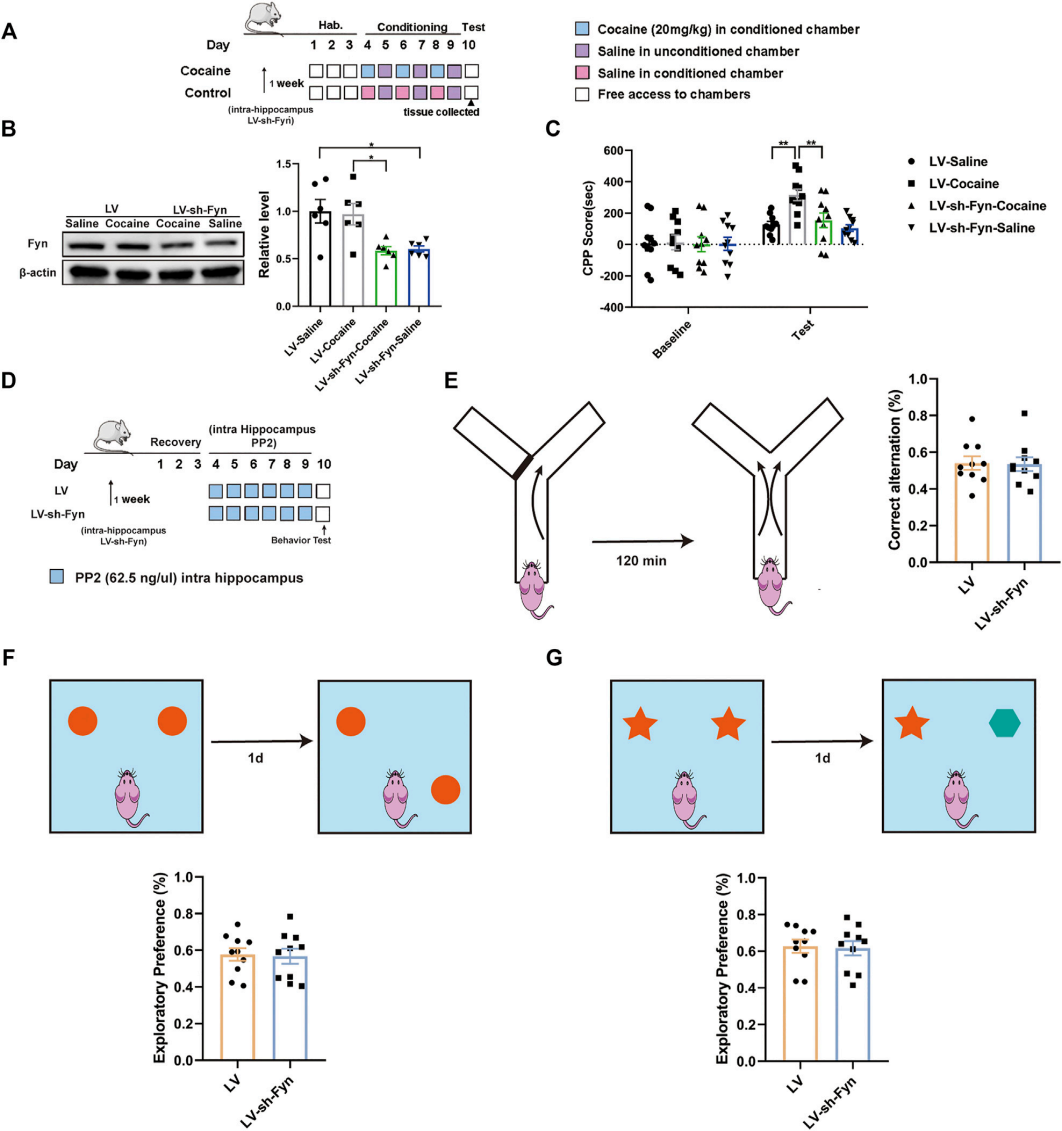
Knock-down PP2 in the hippocampus reduced the cocaine CPP score. **(A)** Experimental timeline for LV-mediated Fyn knock-down in hippocampus 1 week before cocaine CPP test. **(B)** Immunoblotting of Fyn in the hippocampus after LV-mediated Fyn knock-down (*n* = 6 per group). **(C)** Knock-down Fyn significantly attenuated the cocaine CPP score (*n* = 10 per group). **(D)** Experimental timeline for PP2 injection in Fyn knock-down mice. **(E)** There was no significant difference across groups in the Y-maze test after PP2 injection of Fyn knock-down mice (*n* = 10 per group). **(F, G)** Fyn-knock down mice failed to exhibiting exploratory preferences in the novel object position test and novel object recognition test after PP2 administration (*n* = 9 per group). Data are the means ± SEM, *p < 0.05 and **p < 0.01. Hab, habituation.

As PP2 is a nonspecific inhibitor of the Src kinase Fyn, we used a genetic approach to further assess the direct contribution of Fyn to cocaine-associated memory formation. We performed shRNA-mediated knockdown of Fyn in the dorsal hippocampus, and this approach ensured specificity for Fyn and limited the manipulation to the dorsal hippocampus. The timeline of the cocaine CPP procedure is shown in Figure 6A. Notably, the Fyn level was significantly decreased after the injection of LV-sh-Fyn [F _(3, 20)_ = 6.663, *p* < 0.01, one-way ANOVA, Figure 6B]. Compared with the control groups, Fyn knockdown in the dorsal hippocampus substantially attenuated the cocaine-CPP score [F _(3, 36)_ = 8.367, *p* < 0.01, two-way ANOVA, Figure 6C]. We continued to administrate of PP2 into the hippocampus of Fyn knock-down mice (Figure 6D). Notably, no significant difference was observed between Fyn knockdown and control mice in performance on the Y-maze, novel object position, and recognition test (Figures 6E–G), suggesting that neither PP2 nor shRNA-mediated knockdown of Fyn affected short-term working memory and short-term spatial and recognition memory.

Taken together, our data indicate that systemic pharmacological and dorsal hippocampal genetic inhibition of Fyn strongly and selectively suppresses cocaine-associated memory formation and thus represents a promising option to explore for the development of targeted therapies for cocaine addiction.

## Discussion

Previous studies have shown that the hippocampal Src kinase Fyn is involved in cocaine-associated memory formation (Wells et al., 2016) and drug-induced behaviour (Xie et al., 2013; Egervari et al., 2020). In the present study, we explored the mechanism by which Fyn mediates cocaine-associated memory formation. We found that cocaine exposure increases Fyn activity in the hippocampus and that Fyn may regulate Tau expression, which plays an important role in cocaine-associated memory formation (Li et al., 2021). Our results suggested that inactivation of the Src kinase Fyn in the hippocampus significantly disrupts cocaine-induced seeking behaviours in a self-administration paradigm and a cocaine-CPP paradigm without altering locomotor activity. In addition, inhibition of Fyn activity by PP2 reverses the cocaine-induced decrease in Tau expression. Our findings indicated that Fyn activation contributes to cocaine-associated memory formation and that Fyn inhibitors may represent promising therapeutic agents for the treatment of cocaine addiction.

Based on accumulating evidence, the Src kinase Fyn plays a key role in drug addiction, and long-lasting activation of Fyn has been previously illustrated in alcohol addiction (Wang et al., 2007; Wang et al., 2010; Darcq et al., 2014) and cocaine or heroin addiction (Schumann et al., 2009; Egervari et al., 2020). For example, PP2-induced suppression of Src kinases in the dorsal hippocampus inhibits the reinstatement of cocaine-seeking behaviour (Xie et al., 2013) and disrupts cocaine-associated memory reconsolidation, mainly by mediating GluN2A and GluN2B subunit phosphorylation (Wells et al., 2016). Intriguingly, saracatinib (a Src kinase inhibitor) has been shown not only to inhibit alcohol-induced increases in Fyn and GluN2B levels but also to inhibit alcohol self-administration and seeking behaviours in mice. However, further studies are needed to elucidate whether these inhibitors are specific for Fyn. Reports have shown that chromatin accessibility alterations in the postmortem human brain are specific to Fyn among Src kinases and that siRNA-mediated knockdown of Fyn decreases heroin-induced responses (Egervari et al., 2020). Similarly, a PP2 infusion into the dorsal striatum (but not the ventral striatum) also disrupts alcohol-induced GluN2B phosphorylation and reinstatement of alcohol-seeking behaviour (Wang et al., 2010), indicating that Fyn activity in specific brain regions contributes to drug seeking behaviour. In contrast, recent reports have shown the important effect of Fyn activation in the PrL cortex during cocaine self-administration withdrawal on the ability to decrease subsequent cocaine-seeking behaviour. Dephosphorylation and inactivation of GluN2A- and GluN2B-containing NMDA receptors in the PrL cortex is the main target of PP2 administration immediately after cocaine self-administration (Go et al., 2016).

PP2 is a global inhibitor of the Src kinases Fyn, Lyn, Yes, and Lck (Hanke et al., 1996; Bain et al., 2003). It is not a specific inhibitor of Fyn, and the roles of different Src kinase family members must be explored. As different memory types may be modulated by Src kinase family members, further studies are essential for assessing the contributions of specific Src kinases to different memory types (destabilization and reconsolidation) that participate in the extended maintenance of cocaine-associated memories.

*In vivo* studies have shown an important role for activated Fyn in aversive conditioned behaviours, as well as alcohol-CPP, alcohol self-administration, and drug-induced reinstatement of alcohol seeking behaviour in a brain region-specific manner (Schafe et al., 1996; Kojima et al., 2005; Isosaka et al., 2008; Wang et al., 2010). In addition, Fyn activation in the dorsal hippocampus is necessary for contextual fear conditioning (Isosaka et al., 2008). However, Fyn-KO mice show hippocampal long-term potentiation (LTP) induction and spatial learning deficits (Grant et al., 1992), and overexpression of a constitutively active Fyn mutant reinforces excitatory postsynaptic potentials in response to afferent stimulation and reduces the hippocampal LTP induction threshold (Lu et al., 1999). The fact that a Fyn inhibitor potently impairs cocaine-associated memory strongly indicates that Fyn and related networks are important targets for further therapeutic development.

At present, the Src kinase Fyn is an emerging therapeutic target for AD (Nygaard, 2018). According to previous reports, Fyn is a master regulator that interacts with different proteins in the brain and peripheral tissue via its SH2 and SH3 domains, suggesting the regulation of a host of multisignalling pathways (Ohnishi et al., 2011). Recent studies have shown that Aβ-induced Fyn activation alters the total Tau level, although no mechanism for this phenomenon has been identified (Larson et al., 2012). In addition, Fyn activation promotes Tau tyrosine and serine/threonine phosphorylation, suggesting that Fyn activation is involved in mediating both Tau synthesis and phosphorylation (Li and Gotz, 2017). As shown in our studies, treatment with the Fyn inhibitor PP2 reverses the reduction in Tau levels during cocaine-associated memory formation. Previous studies have reported several transcription factor binding sites, including Sp1, GCP, and AP-2, in the Tau promotor region (Heicklen-Klein and Ginzburg, 2000). Sp1 has been shown to be a downstream regulator of MAPK, and MAPK signalling is downstream of Fyn (Xu and Shu, 2007). Based on these results, we infer that the cocaine-induced reduction in Tau levels is related to Sp1 or the suppression of other genes, which requires further investigation. In addition, we used a Fyn inhibitor to study the underlying mechanism by which Fyn modulates Tau expression and measured the level of phosphorylated Fyn after cocaine-associated memory formation. We found that Fyn was activated and the Tau level was decreased. Pretreatment with PP2 inhibited Fyn activity and reversed Tau expression, further disrupting cocaine-associated memory formation. Overall, our data indicate that Tau expression is reduced by Fyn activation, consistent with previous studies (Chen et al., 2013) indicating that Tau levels depend on Fyn activity, especially during cocaine-associated memory formation.

In summary, the present study identified Fyn-mediated regulation of Tau expression in the hippocampus as a novel mechanism that may critically support cocaine-associated memory formation. The underlying mechanism may involve direct increases in Tau levels through the inhibition of Fyn activity. In addition, the present results may further clarify the complex role of the hippocampus in cocaine-associated memory formation and may direct the development of treatments to inhibit drug-associated memory. Finally, based on these results, we propose that Fyn-mediated Tau expression in the hippocampus is necessary for drug-associated memory formation.

## Data Availability

The original contributions presented in the study are included in the article, further inquiries can be directed to the corresponding author.
